# Association Between the Children's Dietary Inflammatory Index (C-DII) and Markers of Inflammation and Oxidative Stress Among Children and Adolescents: NHANES 2015-2018

**DOI:** 10.3389/fnut.2022.894966

**Published:** 2022-05-27

**Authors:** Chuang Zhang, Weirui Ren, Meng Li, Wenbo Wang, Chi Sun, Lin Liu, Yanbin Fang, Lin Liu, Xiaofeng Yang, Xiangjian Zhang, Suolin Li

**Affiliations:** ^1^Department of Pediatric Surgery, The Second Hospital of Hebei Medical University, Shijiazhuang, China; ^2^Department of Gastroenterology, The Third Hospital of Hebei Medical University, Shijiazhuang, China; ^3^Hebei Key Laboratory of Vascular Homeostasis and Hebei Collaborative Innovation Center for Cardio-Cerebrovascular Disease, Shijiazhuang, China

**Keywords:** children's dietary inflammatory index, inflammation, oxidative stress, NHANES, cross-sectional study

## Abstract

**Objectives:**

To explore the association of Children's Dietary Inflammatory Index (C-DII) scores with inflammation and markers of inflammatory factors in children and adolescents.

**Methods:**

Data on dietary nutrient intake, markers of inflammation (ferritin, alkaline phosphatase, C-reactive protein (CRP), absolute neutrophil cell count and lymphocyte count) and oxidative stress (serum bilirubin, albumin, and iron) were available for participants aged 6–19 years (*n* = 1281). Each participant's C-DII score was calculated based on a 24-h diet and recall. Generalized linear models were applied to examine associations between C-DII and markers of inflammation and oxidative stress, while adjusting for covariates. Restricted cubic splines were used to explore the dose-response association of C-DII scores with indicators of inflammatory oxidative stress. Akaike's Information Criterionwas applied to compare the performance of linear and non-linear models.

**Results:**

After adjusting for potential confounders, quantile regression results showed that when comparing C-DII quartile 4 (most pro-inflammatory) and quartile 1 (most anti-inflammatory), lymphocytes, ferritin, CRP were statistically significant differences in serum bilirubin, albumin and serum iron (*P* < 0.05). The C-DII score showed a non-linear relationship with inflammatory oxidative stress indicators. Overweight/obese children and adolescents who ate a high pro-inflammatory diet were more likely to have higher levels of inflammatory cytokines (*P* = 0.002).

**Conclusions:**

The dietary inflammatory index in children is associated with markers of chronic inflammation and oxidative stress. A pro-inflammatory diet resulted in increased serum concentrations of these markers, implying that early dietary interventions have implications for reducing chronic inflammation and oxidative stress in children and adolescents.

## Introduction

Oxidative stress refers to the overloading of the cytoprotective polyphenol system with reactive oxygen species, resulting in oxidative damage to macromolecules, such as DNA, proteins, and lipids (1). Oxidative stress is well-established as the trigger and endpoint of multiple events, including inflammation, hypoxia, and hyperoxia (2). Inflammation is generally divided into acute and chronic inflammation in accordance with a temporal criterion. The primary cells of acute inflammation are mostly neutrophils, while monocytes and lymphocytes are typical immune cells observed during chronic inflammation (3, 4). A strong relationship between chronic low-grade inflammation and oxidative stress has been documented in the literature (5).

An increasing body of evidence suggests that low levels of chronic systemic inflammation and oxidative stress are associated with the development of many chronic diseases, such as diabetes, obesity, cardiovascular disease, cancer, respiratory and musculoskeletal diseases, and impaired neurodevelopment (6–9). Given that children and adolescents have unique nutritional needs and immature immune functions, they are susceptible to infections (10). Accordingly, factors that may affect changes in inflammatory and oxidative stress levels of children and adolescents are currently under study in immunological studies.

Dietary nutrition is a key variable influencing chronic inflammation and oxidative stress status, primarily because daily food intake is a good indicator of a person's dietary inflammatory and oxidative stress potential (11, 12). Indeed, a healthy diet reduces the adverse effects of inflammation and oxidative stress markers in children (13). Growing evidence suggests that dietary patterns or indicators represent markers of inflammatory oxidative stress (14, 15). For example, studies on indicators and dietary patterns, such as the Mediterranean Diet Score, Italian Mediterranean Index, and Healthy Eating Index, have shown that healthier scores are inversely associated with inflammation and indicators of oxidative stress (16, 17).

The Dietary Inflammatory Index (DII) is a new tool to assess the inflammatory potential of an individual's diet; it measures the likelihood that a person's diet causes inflammation (18). In addition, the Children's DII (C-DII) is a new tool developed to assess the diet quality and inflammatory properties in a special child cohort in pediatric practice (19). Overwhelming evidence substantiates a positive correlation between C-DII and inflammatory biomarkers (interleukins 1, 2, and 6; tumor necrosis factor-alpha; and interferon- and soluble vascular cell adhesion molecule-1) (20). DII was developed to characterize an individual's diet's anti- and pro-inflammatory effects. However, few studies have hitherto investigated the role of diet-related inflammation in adolescents (20–24). Since childhood and adolescence represent critical periods of growth and development, the health status during these periods may affect health outcomes in adulthood. Therefore, understanding the intrinsic relationship between the potential inflammatory potential of children's diet and the level of inflammatory oxidative stress has great guiding significance for dietary intervention in early disease and sub-health states.

To the best of our knowledge, few studies have examined the relationship between C-DII scores and biomarkers of inflammation and oxidative stress in children and adolescents. Therefore, the current study sought to assess the relationship of C-DII scores with markers of inflammation [ferritin, alkaline phosphatase (ALP), C-reactive protein (CRP), absolute neutrophil count, and lymphocyte count] and oxidative stress (serum bilirubin, albumin, and iron) among children and adolescents (aged 6–19 years) in the National Health and Nutrition Examination Survey (NHANES) datasets from 2015 to 2018. Furthermore, given that inflammatory oxidative stress levels and C-DII values vary according to the body mass index (BMI), known to be associated with chronic systemic inflammation, BMI was examined as a potential modulator of effects.

## Methods

### Study Population

NHANES is a cross-sectional, nationally representative survey of the US population that is usually carried out over 2 years. The survey respondents are often unorganized, ordinary, and American nationals. Participants are instructed to complete in-depth interviews and quizzes involving precise measurements in medicine and physiology and their laboratory tests. NHANES applies a complex four-step research design scheme to obtain symbolic templates. Since 1999, the NHANES scientific research has been approved by the National Health Statistics Core Organization Verification Consortium and the Ethical Review Board. This secondary analysis of application-identifying information was declared exempt by the Albert Newton Institute for Tissue Verification Consortium. Written consent was obtained from all participants in the NHANES Basic Survey over the age of 12 years and the parents or legal guardians of pediatric clinic participants aged 7–11 years. Secondary analysis of current data information was not permitted without actual written material.

**Figure 1:**
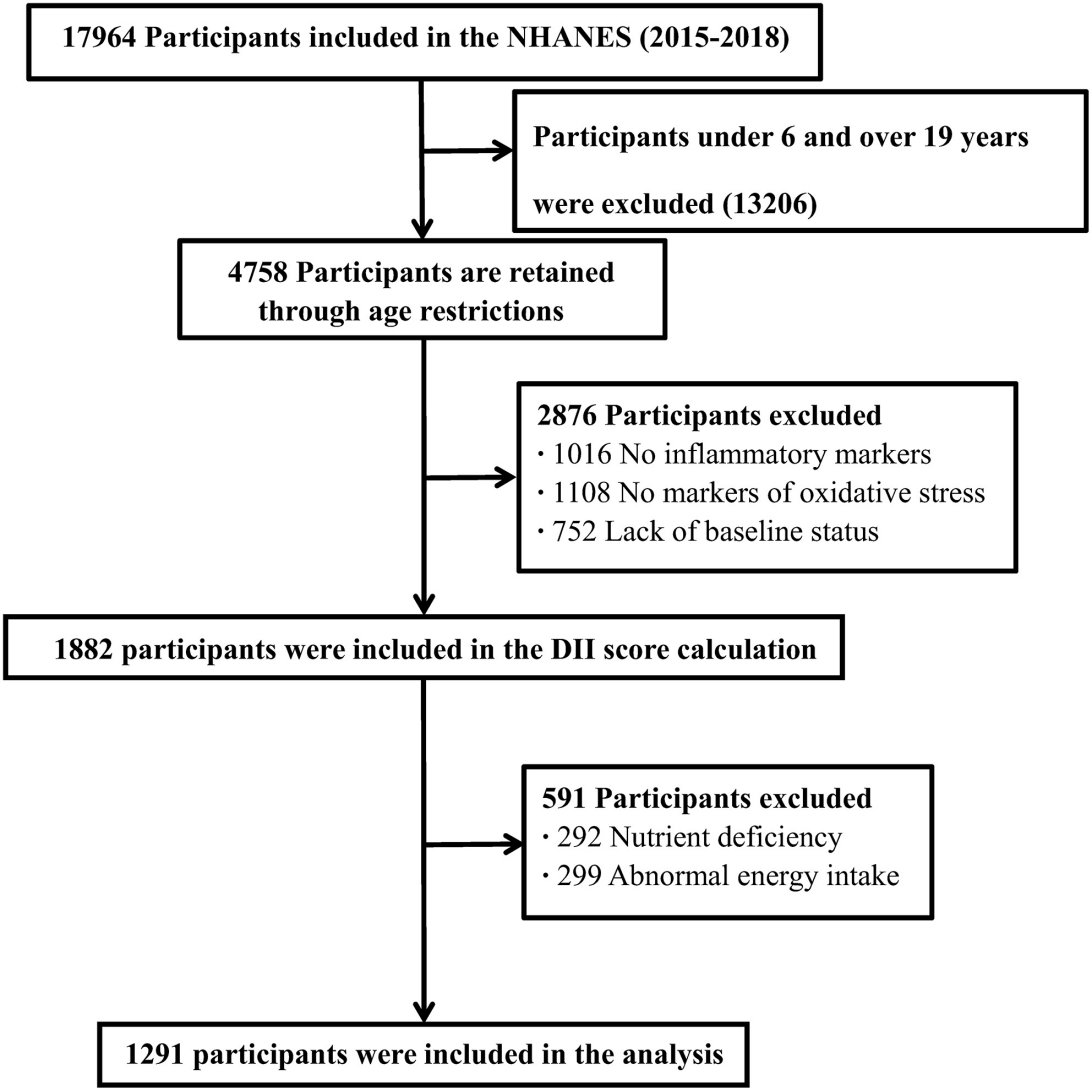
Flow diagram of analytic samples from NHANES 2015-2018.

This study analyzed sociodemographic data, lifestyle factors, inflammatory and oxidative stress indicators, and dietary information data for children and adolescents aged 6–19 years in two NHANES cycles from 2015 to 2018 (Figure 1).

### Outcome Variables

Outcome variables consisted of markers of inflammation and oxidative stress, including ferritin (measured with the Roche Elecsys-17 immunoassay), lymphocyte count (evaluated using automated hematology analyzing devices) (25), absolute neutrophil count (determined using the Beckman Coulter MAXM upon receipt of samples of MECs, as described on the NHANES website) (26), hsCRP [SYNCHRON System(s) High Sensitivity C-Reactive Protein reagent was based on the highly sensitive Near-Infrared Particle Immunoassay rate methodology], and albumin (the DcX800 bichromatic digital endpoint method), serum iron (the DcX800 timed-endpoint method), ALP [a simple reaction wherein ALP acts upon a substrate (p-nitrophenol phosphate, or PNPP) in the presence of magnesium and zinc activators to form a colored product (p-nitrophenol), whose appearance was measured at 450 nm], and bilirubin (coupling with 3,5-chlorophenyl diazonium in the presence of a solubilizing agent in a strongly acidic medium to produce azobilirubin). The latter three variables are also markers of liver disease.

### Exposure Assessment

In NHANES, dietary nutrient intake was assessed by the 24-h dietary recall (24HR) method, whereby professional technicians asked participants about the types and quantities of foods and beverages consumed within 24 h and recorded them in the NHANES computer-assisted dietary survey system. The intake of each food component was then estimated from the University of Texas Food Intake Analysis System and the USDA Survey Nutrient Database. C-DII was calculated using the average nutrient intake on the first day of the 24-h meal recall. For dietary information collected from participants, the validated C-DII method used consisted of 25 components (referred to as food parameters in the DII method). The micronutrients and macronutrients included in the C-DII calculation provided by NHANES were termed food parameters, including carbohydrate, protein, fat, alcohol, fiber, cholesterol, saturated fat, monounsaturated fatty acids, polyunsaturated fatty acids, niacin, thiamin, riboflavin, vitamin B12, vitamin B6, iron, magnesium, zinc, selenium, vitamin A, vitamin C, vitamin E, folic acid, and beta carotene (19).

Calculation of the dietary inflammatory index in children was conducted as previously described in the literature. Briefly, the Z score was calculated by comparing the average and standard deviation of common dietary nutrients with the individual dietary nutrient intake assessed by 24-h dietary review method. The obtained Z score was converted to a percentile value, then the obtained percentile value was doubled, and “1” was subtracted for centralization (from −1 to +1, centered on 0). Finally, the obtained value was multiplied by the inflammation effect score of the corresponding food nutrient provided in the literature to obtain each nutrient's dietary inflammation index score. The dietary inflammation index scores of all food nutrients were added to obtain every individual's overall dietary inflammation index scores.

C-DII calculation formula:


$$\begin{array}{l} \text{Z}score\text{=}[(\text{daily mean intake-global daily mean intake})\text{/standard deviation}]\\ \text{Z}score^{\text{1}}\text{=Z}score\to (\text{converted to a percentile score})\times \;\text{2-1}\\ \text{C-DII=}\sum \text{Z}score^{\text{1}}\times \text{the inflammatory effect}\\ \text{score of each dietary component} \end{array}$$

### Covariates

For regression analysis, we assessed the association with potential confounders, including age (continuous), race/ethnicity (categorical), BMI category, poverty-to-income ratio (PIR, continuous) and cotinine exposure status (categorical). The BMI of children and adolescents aged 2–19 years in NHANES was calculated and stratified into normal weight (BMI <85 percentile) and overweight/obesity (BMI ≥ 85 percentile) (27). PIR was calculated by dividing household income (based on poverty guidelines for household size) by year and state to reflect the socioeconomic status of participants. Race/ethnicity was stratified into non-Hispanic white, non-Hispanic black, Hispanics (Mexican American and other Hispanic), and others. Given that the distribution of serum cotinine was highly right-skewed even after transformation and was detected in only 68% of samples, it was adjusted based on the LOD (0.015 ng/mL) to ensure environmental tobacco exposure was taken into account.

### Statistical Analyses

SAS statistical analysis software (version 9.2, SAS Institute) was used for data analysis. It is well-established that NHANES adopts a complex multi-link design scheme, in which some subgroups are oversampled to improve the accuracy of this same set of data information. A weight calculation was performed on the sample to obtain the results of the marketing promotion to the U.S. population to better adjust for this scheme in the statistical analysis of the data. The MEC sample weight values that matched the 2 survey cycles were matched, and WTMEC2YR (matched to the 2-year mobile exam body weight value) was multiplied by 0.5 (matched to the two phases).

Normally distributed continuous variables were described as the mean ± standard deviation value, while non-parametric variables were described by the median and interquartile range. The student's *t*-test was used to compare groups of normally distributed data; otherwise, the Mann–Whitney test was applied. Categorical variables were presented as percentages and compared by the χ^2^ test. After adjusting for different covariates, weighted multivariable logistic regression was used to examine the relationship between measures of inflammatory oxidative stress and continuous C-DII or quartile C-DII. Model 1 represented the unadjusted model; Model 2 was adjusted for age, sex, race/ethnicity, cotinine exposure status, BMI, and poverty-income ratio; and Model 3 was based on Model 2 and further adjusted for body fat percentage and energy intake. Continuous variables were converted to dichotomous variables based on the median value of the inflammatory oxidative stress indicator, and potential non-linear relationships were examined using restricted cubic splines with three knots in the C-DII range. Non-linearity was assessed using the Wald test.

Stratified analyses were performed to explore effect modification by BMI (BMI ≥ 85th percentile and BMI <85th percentile). The data were reanalyzed during the sensitivity analysis without considering sampling weights, and the relationship between C-DII and inflammatory oxidative stress indicators was further examined by multivariable logistic regression.

All statistical tests were two-sided, and a *P-*value <0.05 was statistically significant. All statistical analyses were performed using R software (R Project for Statistical Computing version 4.0.4).

## Results

### Population Characteristics

The mean C-DII score was +1.02 (SD = 0.68), with values ranging from a maximum anti-inflammatory value of −4.02 to a maximum pro-inflammatory value of 4.42. Participants in the 1st quantile had the lowest inflammation scores (−4.02 to −0.02), and participants in the 3rd and 4th quantiles had the most pro-inflammatory diets (2.11–4.42). Overall, the population (*n* = 1281) was predominantly female (67.4%). In addition to this, the population was predominantly non-Hispanic White (31.6%), mostly cotinine exposed (64.6%), moderate household economic level (mean PIR and standard deviation: 2.09 ± 1.51 years) and higher energy intake (mean energy intake and standard deviation: 1,984.34 ± 959.21 kcal, data not shown).

Table 1 showed the participant characteristic distribution of each quartile of C-DII. In contrast, for subjects in the most anti-inflammatory DII category, the most pro-inflammatory subjects were more likely to be female, younger, non-Hispanic black, have higher rates of overweight or obesity, cotinine exposure, and higher energy intake. No significant differences were observed between C-DII quartiles except for age, sex, BMI, cotinine exposure, and energy intake. At the same time, we observed higher concentrations of CRP, serum bilirubin, albumin, and serum iron in the fourth quantile of DII compared to the first quantile of C-DII (Supplementary Table S1).

**Table 1:** Distribution of characteristics across quartiles of C-DII in national health and nutrition examination surveys, United States, 2015-2018.

**Characteristic**	**Frequency (%) or median (IQR)**				***P*-value**
	**Quartile 1** **(*n =* 320)**	**Quartile 2** **(*n =* 320)**	**Quartile 3** **(*n =* 322)**	**Quartile 4** **(*n =* 319)**	
Age (years)					0.016
	15.0 (8.0–17.0)	15.0 (9.0–17.0)	14.0 (11.0–18.0)	12.0 (9.0–17.0)	
Sex					<0.001
Male	157 (49.1%)	101 (31.6%)	88 (27.3%)	71 (22.3%)	
Female	163 (50.9%)	219 (68.4%)	234 (72.7%)	248 (77.7%)	
BMI (kg/m^2^)					0.026
<85th percentile, % (normal weight)	283 (88.4%)	274 (85.6%)	272 (84.5%)	258 (80.9%)	
≥85th percentile, % (overweight/obese)	37 (11.6%)	46 (14.4%)	50 (15.5%)	61 (19.1%)	
PIR					0.064
<1	89 (27.8%)	80 (25.0%)	100 (31.1%)	98 (30.7%)	
1–3	135 (42.2%)	152 (47.5%)	137 (42.5%)	157 (49.2%)	
>3	96 (30.0%)	88 (27.5%)	85 (26.4%)	64 (20.1%)	
Race/ethnicity					0.321
Mexican American	68 (21.3%)	68 (21.3%)	63 (19.6%)	66 (20.7%)	
Other hispanic	26 (8.1%)	31 (9.7%)	28 (8.7%)	30 (9.4%)	
Non-hispanic white	104 (32.5%)	103 (32.2%)	104 (32.3%0	94 (29.5%)	
Non-hispanic black	47 (14.7%)	62 (19.4%)	64 (19.9%)	77 (24.1%)	
Other/multi-racial	75 (23.4%)	56 (17.5%)	63 (19.6%)	52 (16.3%)	
Cotinine exposure status					0.007
Exposed (>0.015 ng/ml)	125 (39.1%)	124 (38.8%)	117 (36.3%)	88 (27.6%)	
Unexposed (≤ 0.015 ng/ml)	195 (60.9%)	196 (61.3%)	205 (63.7%)	231 (72.4%)	
Percent body fat, %					0.002
	29.3 (22.2–34.9)	32.9 (26.3–39.4)	32,4 (27.45–38.0)	34.9 (29.0–39.9)	
Energy intake (kcal)					<0.001
	1,214.0 (890.0–1,654.0)	1,643.0 (1,323.8–2,035.0)	1,934.5 (1,557.0–2,463.0)	2,634.5 (2,038.3–3,384.5)	

*E-DII quartile ranges: quartile 1: −5.46 to −0.06; quartile 2: −0.05, 1.25; quartile 3: 1.26 to 2.25; quartile 4:2.26 to 2.58. C-DII, children's dietary Inflammatory Index; BMI, body mass index; PIR, Ratio of family income to poverty; IQR, interquartile range*.

### Association Between C-DII and Markers of Inflammation and Oxidative Stress

After adjusting for different covariates, significant associations between C-DII and lymphocyte count, ferritin, and CRP were found among inflammatory markers in unadjusted logistic regression models and multivariable logistic regression models. Among oxidative stress indicators, significant positive associations were also observed for bilirubin and albumin (Table 2).

**Table 2:** β Values of C-DII score in linear regression models after adjusting for different covariates.

	**Model 1**	***P*-value**	**Model 2**	***P*-value**	**Model 3**	***P*-value**
**Markers of chronic inflammation**						
Neutrophil count	−0.02 (−0.08,0.05)	0.422	0.02 (−0.05,0.08)	0.286	0.01 (−0.06,0.07)	0.312
Lymphocyte count	0.08 (0.02,0.16)	0.001	0.06 (0.01,0.12)	0.008	0.07 (0.02,0.15)	0.006
Ferritin	0.15 (0.06,0.28)	0.007	0.12 (0.04,0.21)	0.012	0.11 (0.02,0.19)	0.015
Alkaline phosphatase	0.12 (−0.05,0.22)	0.186	0.10 (−0.02,0.18)	0.102	0.08 (−0.01,0.16)	0.082
CRP	0.18 (0.08, 0.32)	0.004	0.16 (0.07,0.26)	0.005	0.17 (0.08,0.28)	0.002
**Markers of oxidative stress**						
Serum iron	0.05 (−0.02,0.11)	0.135	0.08 (−0.01,0.15)	0.086	0.06 (−0.02,0.12)	0.092
Bilirubin	0.08 (0.01,0.17)	0.042	0.07 (0.01,0.18)	0.036	0.09 (0.02,0.20)	0.026
Albumin	0.06 (−0.02,0.12)	0.065	0.08 (0.00,0.15)	0.048	0.12 (0.05, 0.18)	0.032

*Model 1, unadjusted model; Model 2, adjusted for age, sex, race/ethnicity, cotinine exposure status, BMI and poverty-income ratio; Model 3, adjusted for model 2 plus body fat percentage and energy intake*.

**Figure 2:**
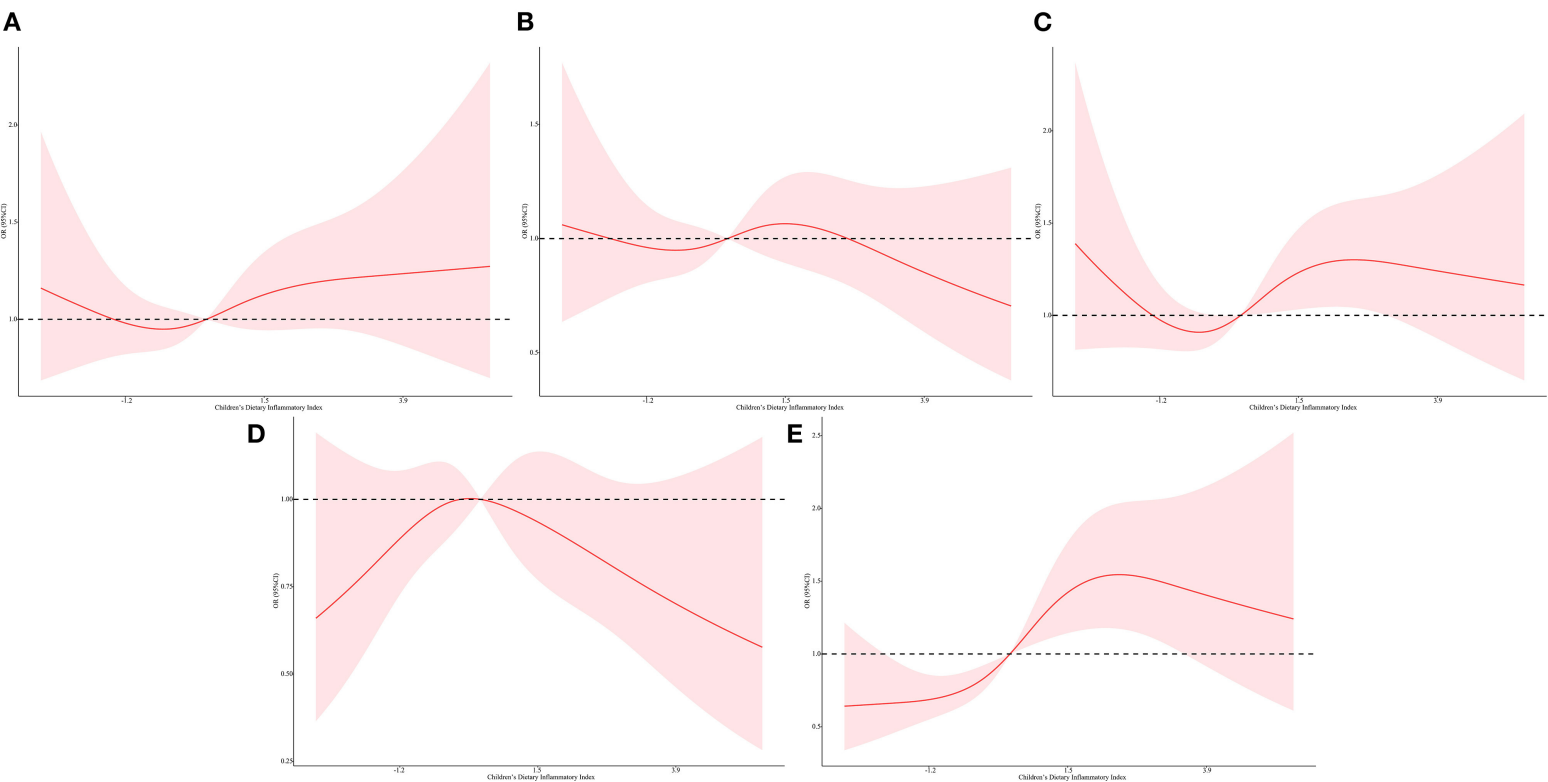
Restricted cubic spline regressions of C-DII and the inflammatory markers after adjusting for different covariates in model 3 for **(A)** lymphocyte count, **(B)** neutrophil count, **(C)** ferritin **(D)** CRP, **(E)** alkaline phosphatase. The red line and area represent the estimated OR values and their corresponding 95% CI. Model 3 adjusted for age, sex, race/ethnicity, cotinine exposure status, BMI, poverty-income ratio, body fat percentage and energy intake.

**Figure 3:**
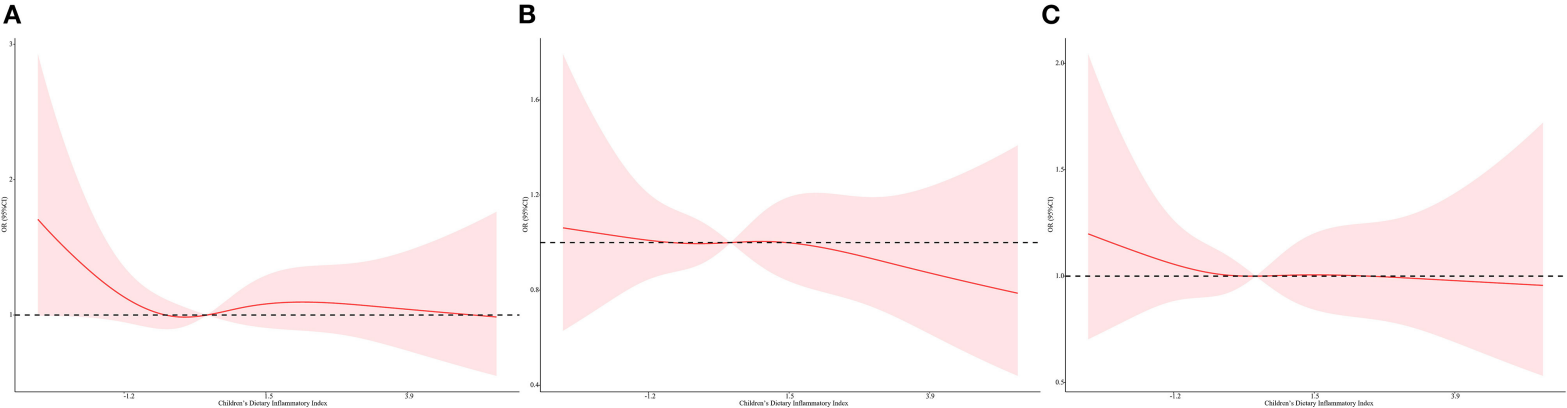
Restricted cubic spline regressions of C-DII and the oxidative stress markers after adjusting for different covariates in model 3 for **(A)** serum bilirubin, **(B)** albumin, **(C)** serum iron. The red line and area represent the estimated OR values and their corresponding 95% CI. Model 3 adjusted for age, sex, race/ethnicity, cotinine exposure status, BMI, poverty-income ratio, body fat percentage and energy intake.

In the restricted cubic spline regression, we found a non-linear relationship between C-DII score and inflammatory oxidative stress index after adjusting for different covariables, and most of them had a significant non-linear association (Figures 2, 3, Supplementary Figures S1–S4). The level of inflammatory oxidative stress increased significantly before c-DII score was 0.15, and the score tended to decrease after 0.15. C-DII scores were then divided into quartiles of categorical variables, with individuals in the lowest quartile of C-DII considered an anti-infective diet, and individuals in the second quartile considered a neutral diet. Participants in the third C-DII quartile and the largest C-DII quartile were considered to have weak and pro-inflammatory diets.

**Figure 4:**
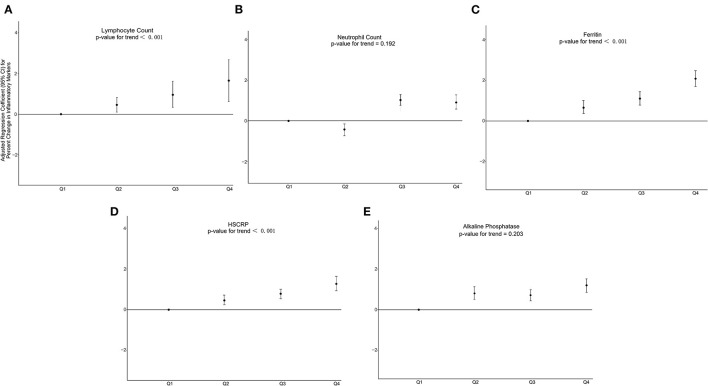
Percent change in inflammatory markers associated with increasing C-DII among study participants in model 3 for **(A)** lymphocyte count, **(B)** neutrophil count, **(C)** ferritin **(D)** CRP, **(E)** alkaline phosphatase. Model 3 adjusted for age, sex, race/ethnicity, cotinine exposure status, BMI, poverty-income ratio, body fat percentage and energy intake.

**Figure 5:**
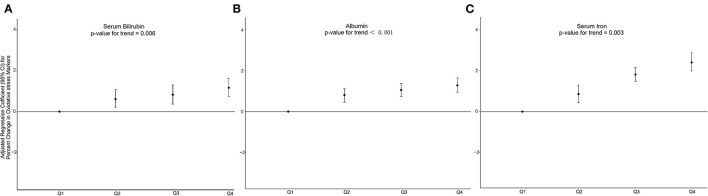
Percent change in oxidative stress markers associated with increasing C-DII among study participants in model 3 for **(A)** serum bilirubin, **(B)** albumin, **(C)** serum iron. Model 3 adjusted for age, sex, race/ethnicity, cotinine exposure status, BMI, poverty-income ratio, body fat percentage and energy intake.

To explore the non-linear relationship between C-DII scores and indicators of inflammatory oxidative stress, individuals in the first C-DII quartile were set as reference. We found that, after adjusting for different covariates, individuals in the fourth C-DII quartile had higher Inflammation (lymphocytes, ferritin, and CRP) and oxidative stress levels (serum iron, bilirubin, and albumin), trend tests showed that these indicators of inflammation and oxidative stress were significantly positively correlated with C-DII (*P* <0.05) (Figures 4, 5, Supplementary Figures S5–S8). The AIC values and Pseudo-R2 of different patterns are shown in Table 3. The AIC values for the C-DII quartile-limited cubic spline regression solid model and the logistic regression model were lower than the AIC values for the logistic regression model for persistent C-DII.

**Table 3:** AIC values and pseudo R^2^ in different models.

	**Model 1**		**Model 2**		**Model 3**	
	**AIC**	**Pseudo-R^**2**^**	**AIC**	**Pseudo-R^**2**^**	**AIC**	**Pseudo-R^**2**^**
Linear model	3,086.72	0	2,265.18	0.28	2,257.62	0.30
Non-linear model	3,080.15	0	2,258.06	0.28	2,248.27	0.30
Quartile model	3,076.62	0	2,252.65	0.29	2,242.16	0.30

*Model 1, unadjusted model; Model 2, adjusted for age, sex, race/ethnicity, cotinine exposure status, BMI and poverty-income ratio; Model 3, adjusted for model 2 plus body fat percentage and energy intake. AIC, Akaike's Information Criterion value*.

### Subgroup Analyses

The interaction results showed that the *p*-values of the interaction terms between C-DII and BMI are 0.06, which may indicate that there is an interaction between C-DII and BMI (data not shown). According to the results of BMI stratification, compared with normal-weight children and adolescents, we found that the level of inflammatory oxidative stress increased significantly with the increase of C-DII score quantiles in overweight or obese children and adolescents. A significant association between C-DII and CRP was only observed in normal-weight children and adolescents (*P* <0.05) (Supplementary Figure S9–S11).

### Sensitivity Analyses

We reanalyzed the data by performing limited cubic spline regression and multiple logistic regression without taking into account the sampling weight values. A clear non-linear relationship (*P* = 0.026) between C-DII scores and markers of inflammatory cytokines was also observed in the unweighted computational data information (Supplementary Figure S12). Also, nonlinear models have better fit than linear models (Supplementary Table S2).

## Discussion

In the present study, we examined the associations between dietary inflammatory potential and markers of inflammatory oxidative stress in children and adolescents. We found significant correlations between C-DII and markers of inflammation, immunity, oxidative stress, and liver injury (*P* <0.05). Subgroup and sensitivity analyses performed to validate the association between C-DII scores and markers of inflammatory oxidative stress showed that the non-linear model provided a better fit than the linear model. The subgroup analysis further showed a more significant positive association between C-DII and markers of inflammatory oxidative stress in overweight/ obese children and adolescents than in normal-weight children and adolescents.

It is widely acknowledged that diet affects inflammation levels, but few relevant studies have assessed how diet affects markers of inflammation among children and adolescents. One of the theories is that a pro-inflammatory diet may increase levels of inflammatory cytokines by affecting oxidative stress and immune mechanisms (28). Ample evidence substantiates that phagocytes synthesize oxygen free radicals and release them into structures after ingesting a pro-inflammatory meal. Importantly, oxygen free radicals that drive somatic inflammatory factors are often associated with increased inflammation, suggesting that a pro-inflammatory diet can lead to inflammation in the plasma (29, 30). It is well-recognized that diets in Western countries can cause high postprandial blood sugar and high blood lipids (31, 32). Indeed, diet is one of the strongest environmental influences on chronic systemic inflammation. Dietary combinations that contain fresh fruits, vegetables and fruits, whole grains, and fingerlings have been associated with moderate systemic inflammation. In contrast, diets characterized by high consumption of total body fat and saturated fatty acids, protein, simple sugars, and carbohydrates (such as Western diets) were associated with higher levels of circulatory inflammatory biomarkers (33). Changes in gut microbiota caused by different dietary patterns may account for changes in circulating inflammation levels. Mounting evidence suggests that dietary habits can further regulate the composition of gut microbiota by affecting inflammatory levels in the body. A pro-inflammatory diet such as the Western diet is associated with lower bacterial diversity. In contrast, anti-infective diets characterized by increased intake of fresh fruits, vegetables and fruits, whole grains, legumes, and dried fruits, increase gut microbial diversity (34).

Our research shows that the C-DII scores reflected an increased risk of elevated inflammatory markers, including reticulocytes, ferritin, and CRP. Indeed, prolonged exposure to pro-inflammatory stimuli can alter the body's overall balance, resulting in additional structural damage. The number of reticulocytes may increase with prolonged inflammation, resulting in further increases in pro-inflammatory cytokines and their ozone and other molecular structures that can sustain inflammation (28). It has been shown that increased lymphocytes can lead to chronic diseases, especially cerebrovascular disease (35). For example, the accumulation of excessive reticulocytes can cause the walls of blood vessels to thicken. At the same time, increased accumulation of reticulocytes can increase inflammation in the affected area, causing further damage (36). In recent years, ferritin has been extensively used as an inflammatory marker in childhood obesity research (37). However, it is well-established that the ferritin/transferrin saturation ratio is a more accurate indicator of ferritin values because of the interference of changes in systemic iron status. Although serum ferritin is often used clinically to assess the systemic iron status and the risk of anemia, ferritin is also an acute-phase protein produced in the body's inflammatory response to infection and injury, similar to CRP. The relationship between C-DII and ferritin has been validated in experimental scientific studies, which showed that dietary nutrients stimulate the release of inflammatory cytokines from human squamous epithelial cells, human umbilical vein endothelial cells, and rat bronchi (38). Elevated CRP serum protein concentration values are commonly used as inflammatory biomarkers associated with various systemic diseases. As an acute phase reactant, CRP increases significantly with somatic cell damage or infection (39, 40).

In this study, we used serum bilirubin, iron, and albumin as biomarkers of oxidative stress. Our results showed that C-DII was significantly positively correlated with oxidative stress indicators. It is widely acknowledged that bilirubin is a biological reductant of endogenous antioxidant activity and a scavenger of reactive oxygen species. Moreover, bilirubin is a product of hemoglobin catabolism, catalyzed by heme oxygenase to decompose heme into carbon monoxide, iron, and bilirubin, produced by biliverdin reductase. Bilirubin can be excreted from the body mainly in the form of pyran through the formation of oxidative metabolites. Both bilirubin and pyran have antioxidant activity and are hence used as biomarkers to assess the level of oxidative stress (41, 42). In addition, a significant association has been documented with other commonly known markers of oxidative stress, such as malondialdehyde (43). It has long been thought that bilirubin is a marker of oxidative stress used to predict several common diseases in children and adolescents (44, 45). Iron is one of the common biological essential trace elements, which plays many critical cellular functions in all organisms while catalyzing the formation of potentially toxic free radicals. A large body of evidence substantiates the potential additive effect of oxidative stress as iron stores increase, further exacerbating disease susceptibility and responses to infection and inflammation (46, 47). The observed increase in iron concentration with C-DII in our study suggests that an inflammatory diet may lead to inflammatory oxidative stress in the body (especially the liver).

Moreover, studies have shown that consumption of a pro-inflammatory diet during adolescence is associated with changes in cardiometabolic risk factors, particularly systolic blood pressure, diastolic blood pressure, and BMI, and higher C-DII scores are directly associated with pro-inflammatory adipokines (22, 23). Indeed, childhood and adolescence are important stages of growth and development, and maternal, paternal, familial and individual risk factors have varying degrees of influence on the C-DII score. Furthermore, early childhood interventions that promote healthy lifestyle behaviors and feeding habits at home and in parenting in children and their parents may help reduce dietary inflammation and associated obesity risk in children (48). Therefore, new public health policies should be implemented to promote healthy eating habits during childhood to prevent premature systemic inflammation and adverse health effects later in life.

Our findings further suggest that the association of C-DII with inflammatory and oxidative stress markers was more pronounced in the overweight and obese subgroup of children and adolescents. In this regard, current evidence suggests that obesity mediates the occurrence of many diseases through low-grade inflammation and plays an essential role in disease pathogenesis (49). During obesity, circulating pro-inflammatory cytokines increase in response to chronic stress leading to increases in inflammatory oxidative stress biomarkers through various actions, including disruption of normal cell division processes and signal transduction (50). Furthermore, diet-induced inflammation was significantly associated with the overweight risk for obesity-related obesity (FTO) gene polymorphism (51). These changes all contribute to a chronic pro-inflammatory state in obese individuals. A valid assumption is that children with high BMI and high inflammation are more vulnerable to harm from a pro-inflammatory diet.

This study evaluated the potential association between C-DII and common biomarkers of oxidative stress and inflammation based on a large sample size (*n* = 1,281). We further explored the possible regulatory effects of dietary intervention on inflammatory oxidative stress levels in children and adolescents. Stratification and quartile analysis were conducted to increase the reliability of our findings. However, several limitations and shortcomings were present in this study. First of all, given the cross-sectional study design, the chronological order and causality could not be determined. Besides, the included data were from a single time point, making it difficult to draw conclusions about the long-term effects of dietary inflammation on markers of inflammatory oxidative stress. High C-DII scores in childhood may contribute to health problems in adulthood that could not be examined in the present study, at least not without complex data associations over the long term. This issue is indeed an inherent problem in studies that examine children. Moreover, only one 24-h dietary recall was used to calculate C-DII. A 24HR may not be able to explain daily changes in dietary intake, which may lead to inaccurate estimates. In addition, potentially confounding variables, such as puberty, were not included in the survey. Furthermore, although C-DII was previously validated using NHANES, its application in other populations is limited, and it has not been validated in other countries or specific subgroups. Further research is indeed warranted to fully characterize its utility.

## Conclusions

The study found that a pro-inflammatory diet was associated with higher levels of inflammatory cytokines, especially in overweight or obese children and adolescents. Elevated levels of inflammatory oxidative stress were associated with several chronic diseases. Given that pro-inflammatory diets are significantly associated with elevated markers of inflammatory oxidative stress, they may impact chronic diseases by affecting levels of inflammatory oxidative stress. However, this particular hypothesis was not explored in the current study. Accordingly, future longitudinal studies are needed to validate how pro-inflammatory diets affect markers of inflammatory oxidative stress and chronic disease pathways.

## Data Availability Statement

The original contributions presented in the study are included in the article/Supplementary Material, further inquiries can be directed to the corresponding author/s.

## Ethics Statement

The studies involving human participants were reviewed and approved by the NCHS Ethic Review Board. Written informed consent to participate in this study was provided by the participants' legal guardian/next of kin.

## Author Contributions

SL and CZ: project development, data collection, management, and analysis, and manuscript writing. WR, CS, LL (6th author), and ML: project development, data management and analysis, and manuscript writing and editing. WW, YF, and LL (8th author): data collection, management, and analysis. XY and XZ: data analysis and manuscript editing. All authors contributed to the article and approved the submitted version.

## Funding

This project partly supported Public walfare research and special funds were received from The National Health and Family Planning of China (201402007).

## Conflict of Interest

The authors declare that the research was conducted in the absence of any commercial or financial relationships that could be construed as a potential conflict of interest. The reviewer YM declared a shared affiliation with the authors to the handling editor at the time of review.

## Publisher's Note

All claims expressed in this article are solely those of the authors and do not necessarily represent those of their affiliated organizations, or those of the publisher, the editors and the reviewers. Any product that may be evaluated in this article, or claim that may be made by its manufacturer, is not guaranteed or endorsed by the publisher.
