# Supplementary material for: Association Between the Children's Dietary Inflammatory Index (C-DII) and Markers of Inflammation and Oxidative Stress Among Children and Adolescents: NHANES 2015-2018
Source: Front Nutr. 2022 May 27;9:894966. doi: 10.3389/fnut.2022.894966 (PMC9195621; doi:10.3389/fnut.2022.894966)
Supplement: Supplementary file 1 [file Data_Sheet_1.DOCX]

**Table S1** Inflammation and oxidative stress index by DII Quartiles, National Health and Nutrition Examination Survey (NHANES), 2015–2018.

| Immune Marker | Quartile 1 (n=732) | Quartile 2 (n=732) | Quartile 3 (n=732) | Quartile 4 (n=732) | P-value |
| --- | --- | --- | --- | --- | --- |
| Lymphocyte Count (1000 per uL) | 2.1(1.8-2.7) | 2.1(1.7-2.7) | 2.2(1.8-2.7) | 2.2(1.7-2.7) | 0.834 |
| Neutrophil Count (1000 per uL) | 4.1(3.1-5.2) | 4.1(3.1-5.1) | 4.0(3.1-5.2) | 4.1(3.1-5.3) | 0.304 |
| HSCRP | 2.1(0.9-4.5) | 2.2(1.0-5.2) | 2.4(1.0-5.3) | 2.8(1.0-6.1) | ＜0.001 |
| Serum Bilirubin (mg/dL) | 0.4(0.3-0.6) | 0.4(0.3-0.6) | 0.4(0.3-0.6) | 0.4(0.3-0.5) | 0.001 |
| Albumin (g/L) | 41(39-43) | 41(39-43) | 41(38-43) | 40(38-42) | ＜0.001 |
| Alkaline Phosphatase | 20(14-40) | 21(14-45) | 21(14-49) | 22(14-48) | 0.337 |
| Iron (μmol/L) | 14.4(10.4-18.4) | 13.8(10.6-18.1) | 14.1(10-18.4) | 13.3(9.7-17.4) | 0.001 |
| Ferritin (μg/L) | 81.9(36.8-165.8) | 83.5(36.5-173.8) | 80.7(31.5-164.8) | 76.8(32.0-164.8) | 0.432 |

**Table S2** AIC values in different subgroup and unweighted data models.

|  | Linear model | Non-linear model |
| --- | --- | --- |
| Normal weight subgroup | 2086.28 | 2085.82 |
| Overweight/obese subgroup | 1906.15 | 1905.98 |
| Unweighted data | 2415.87 | 2412.62 |

Note: AIC, Akaike’s Information Criterion value.


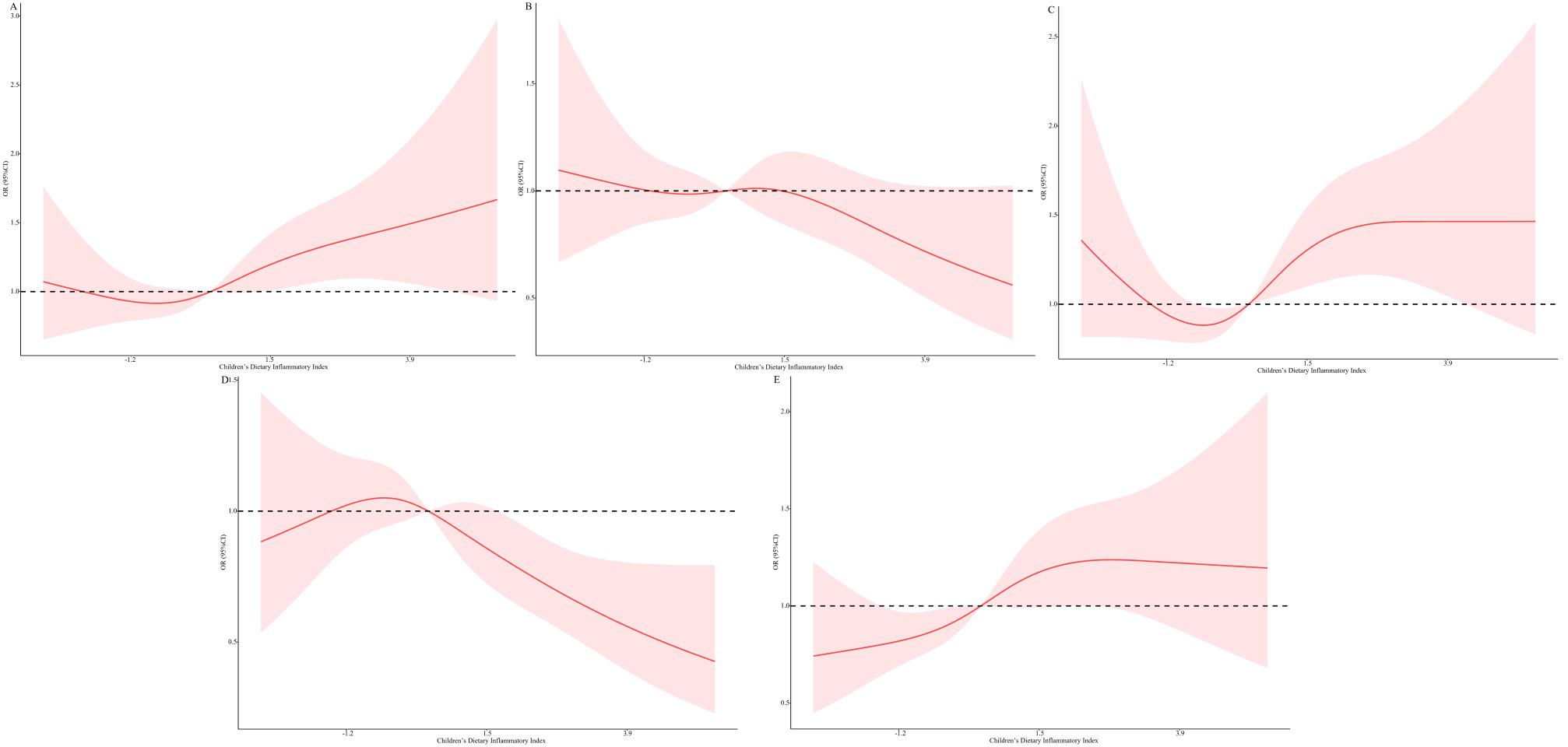


**Figure S1** Restricted cubic spline regressions of C-DII and the inflammatory markers after adjusting for different covariates in model 1 for (a) lymphocyte count, (b) neutrophil count, (c) ferritin (d) CRP, (e) alkaline phosphatase. The red line and area represent the estimated OR values and their corresponding 95% CI. Model 1, unadjusted model.


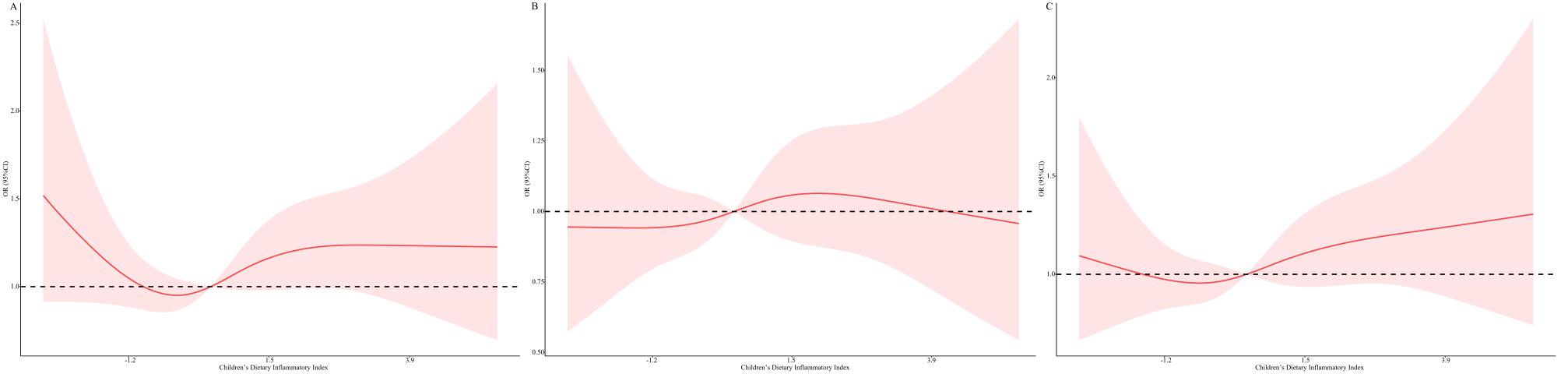


**Figure S2** Restricted cubic spline regressions of C-DII and the oxidative stress markers after adjusting for different covariates in model 1 for (a) serum bilirubin, (b) albumin, (c) serum iron. The red line and area represent the estimated OR values and their corresponding 95% CI. Model 1, unadjusted model.


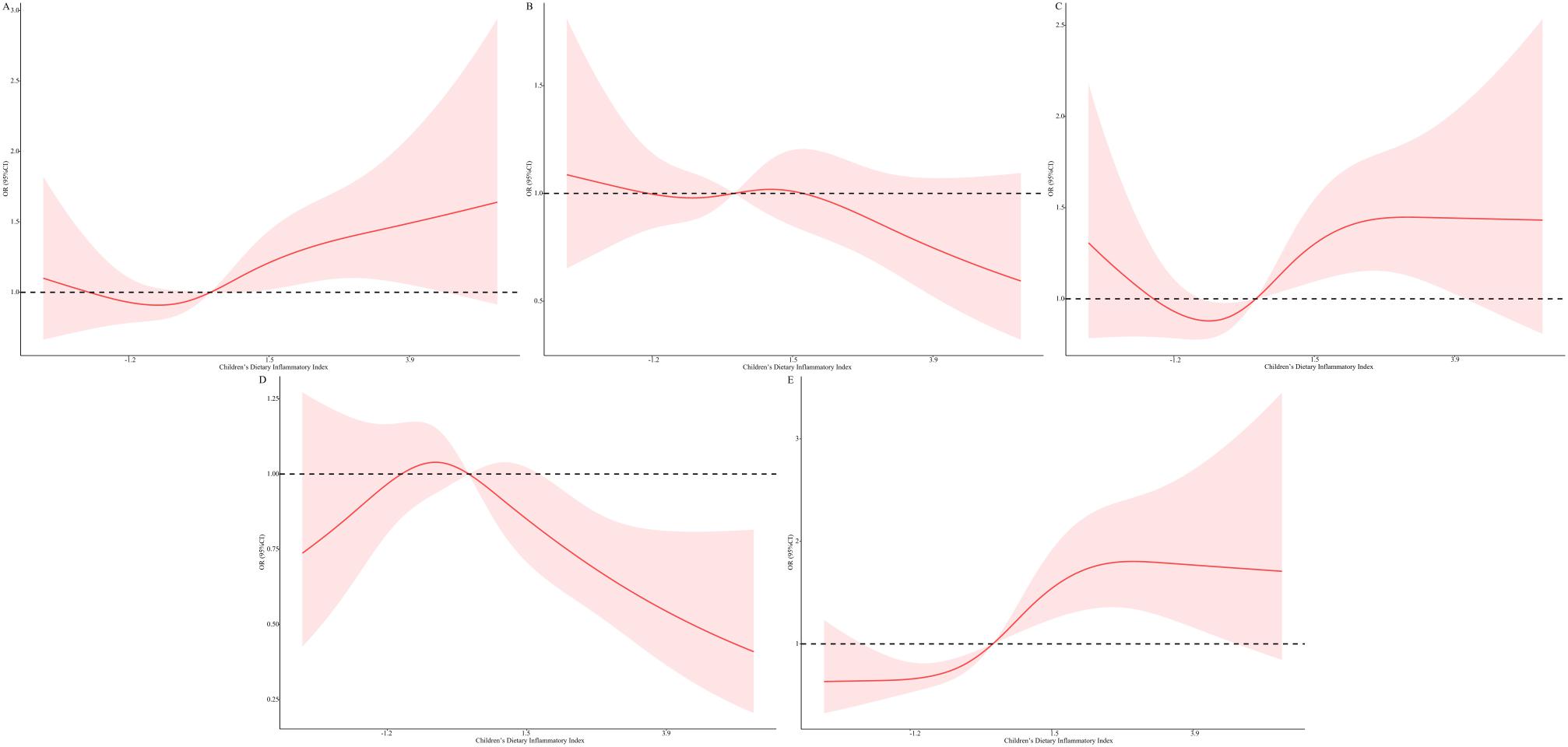


**Figure S3** Restricted cubic spline regressions of C-DII and the inflammatory markers after adjusting for different covariates in model 2 for (a) lymphocyte count, (b) neutrophil count, (c) ferritin (d) CRP, (e) alkaline phosphatase. The red line and area represent the estimated OR values and their corresponding 95% CI. Model 2 adjusted for age, sex, race/ethnicity, smoking status, BMI, energy intake and poverty-income ratio.


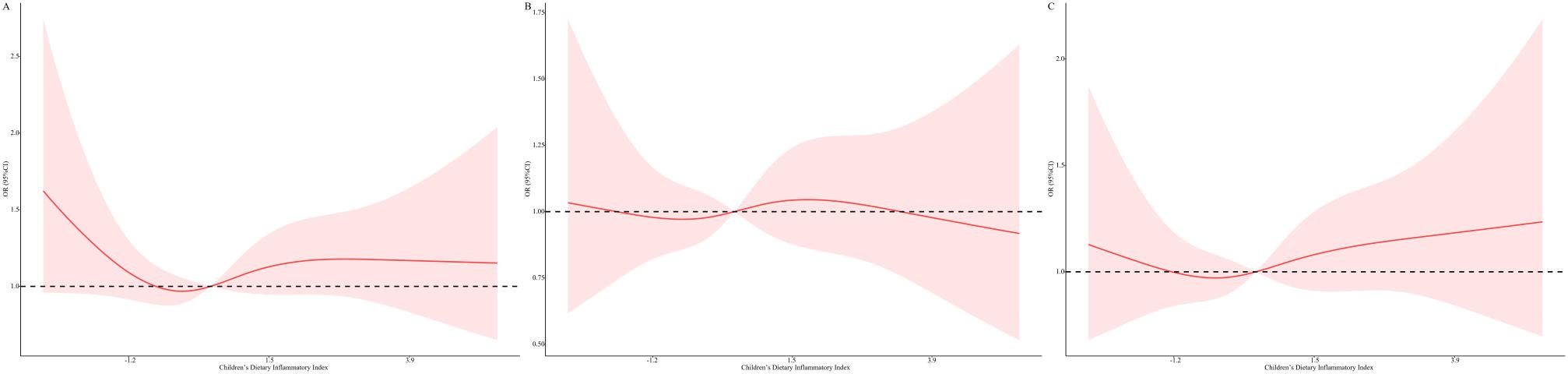


**Figure S4** Restricted cubic spline regressions of C-DII and the oxidative stress markers after adjusting for different covariates in model 2 for (a) serum bilirubin, (b) albumin, (c) serum iron. The red line and area represent the estimated OR values and their corresponding 95% CI. Model 2 adjusted for age, sex, race/ethnicity, smoking status, BMI, energy intake and poverty-income ratio.


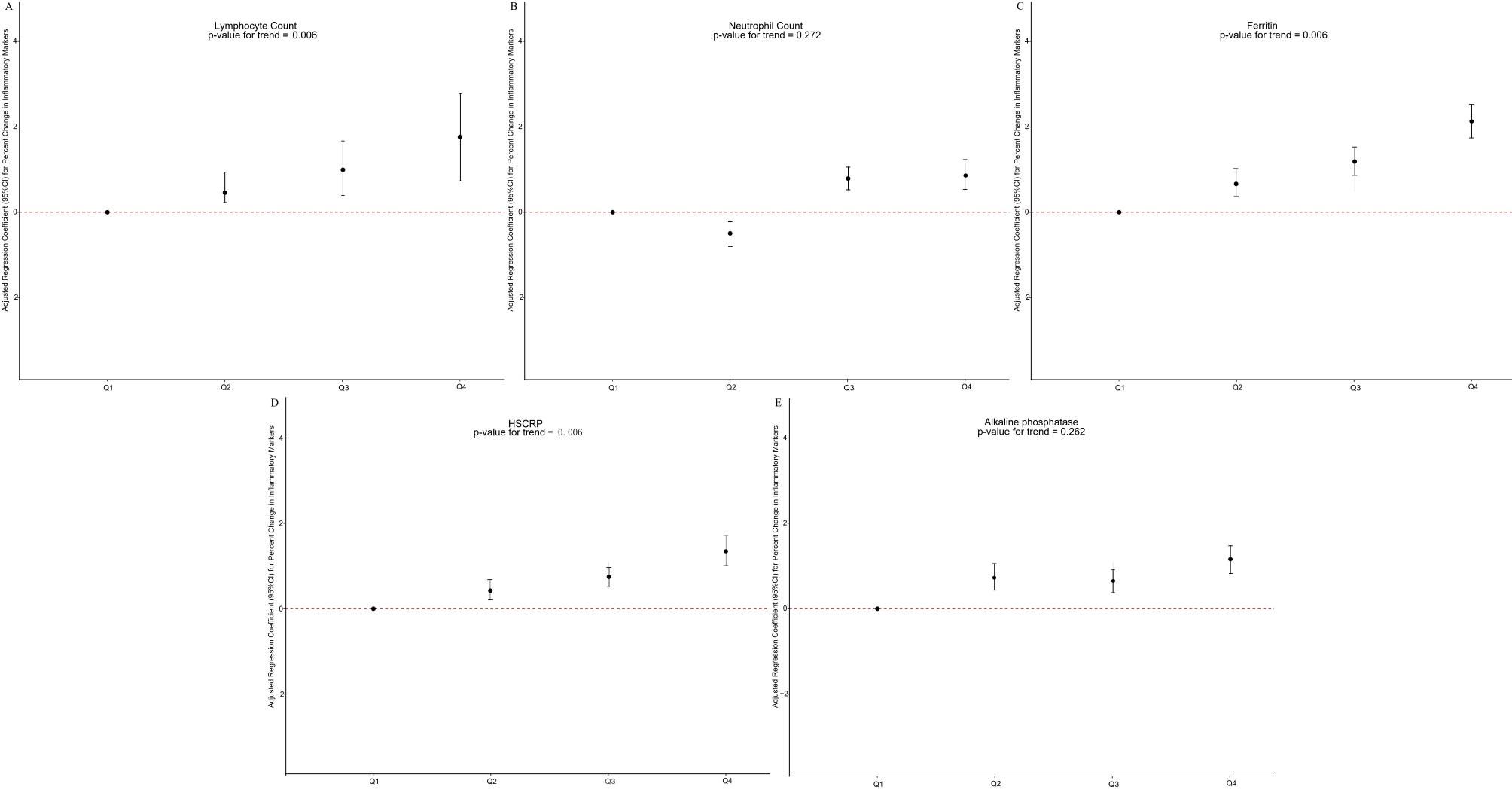


**Figure S5** Percent Change in Inflammatory Markers Associated with Increasing C-DII among Study Participants in model 1 for (a) lymphocyte count, (b) neutrophil count, (c) ferritin (d) CRP, (e) alkaline phosphatase. Model 1, unadjusted model.


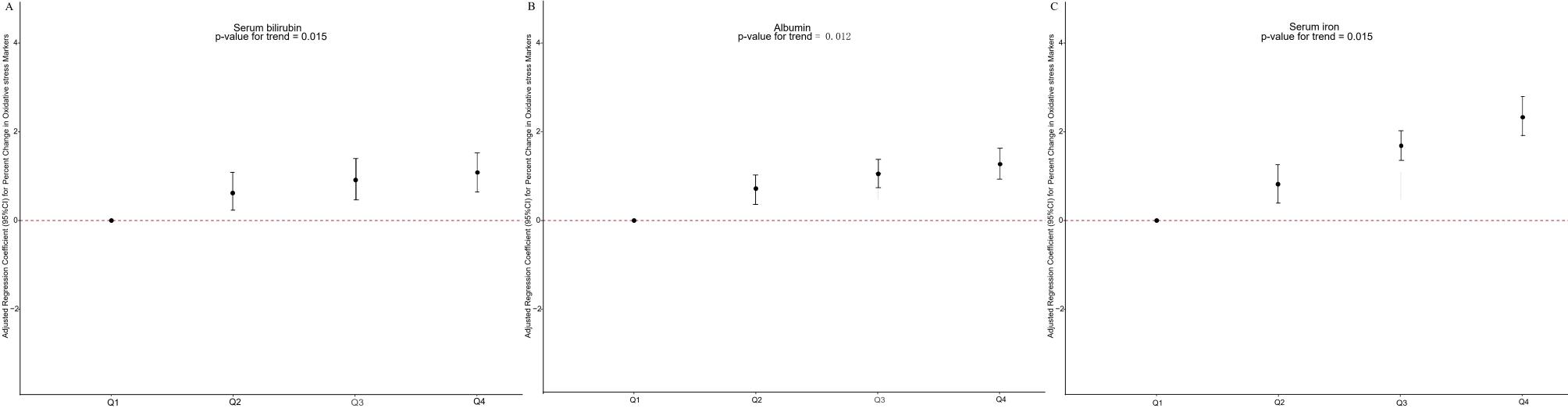


**Figure S6** Percent Change in Oxidative Stress Markers Associated with Increasing C-DII among Study Participants in model 1 for (a) serum bilirubin, (b) albumin, (c) serum iron. Model 1, unadjusted model.


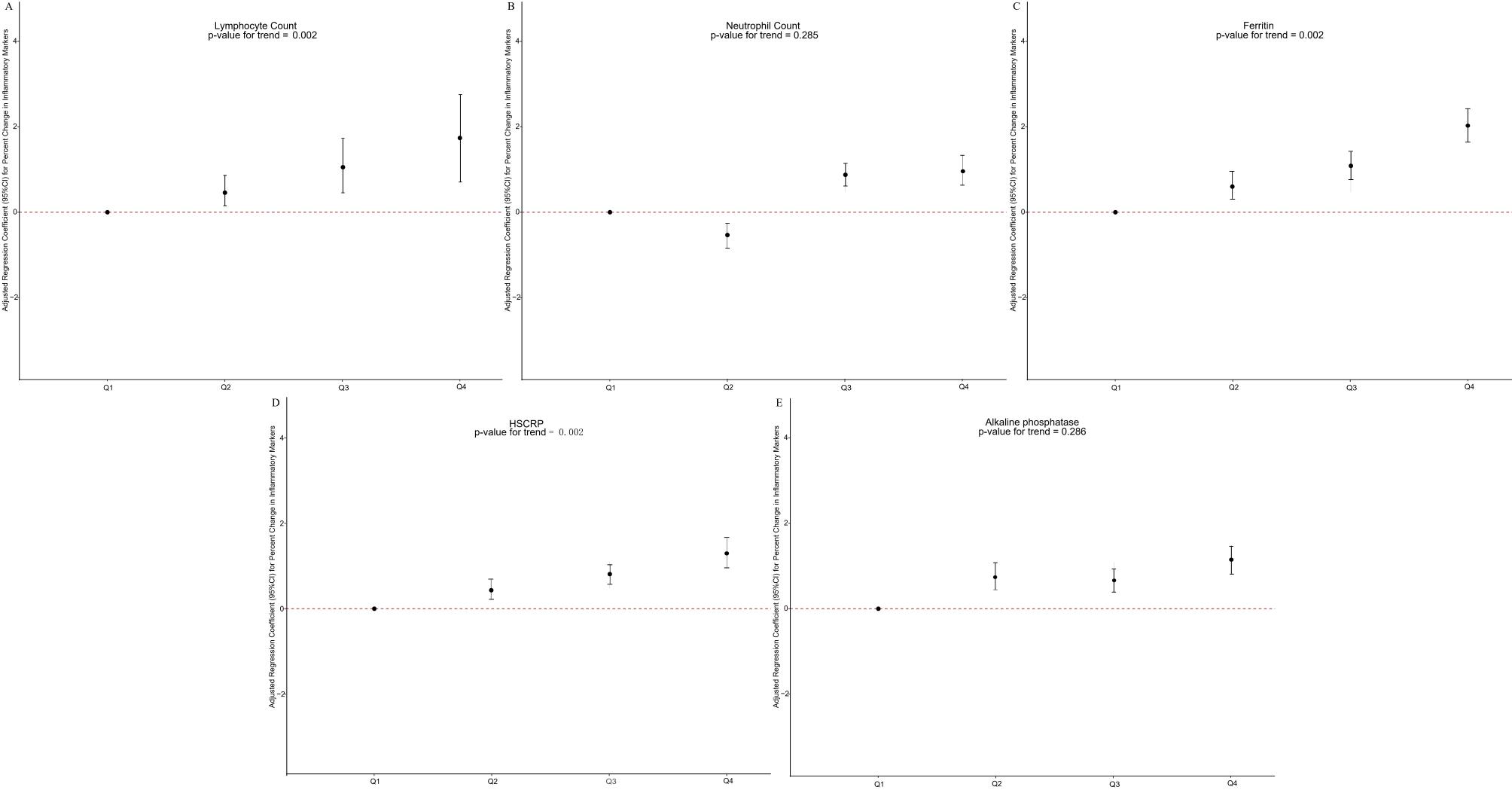


**Figure S7** Percent Change in Inflammatory Markers Associated with Increasing C-DII among Study Participants in model 2 for (a) lymphocyte count, (b) neutrophil count, (c) ferritin (d) CRP, (e) alkaline phosphatase. Model 2 adjusted for age, sex, race/ethnicity, smoking status, BMI, energy intake and poverty-income ratio.


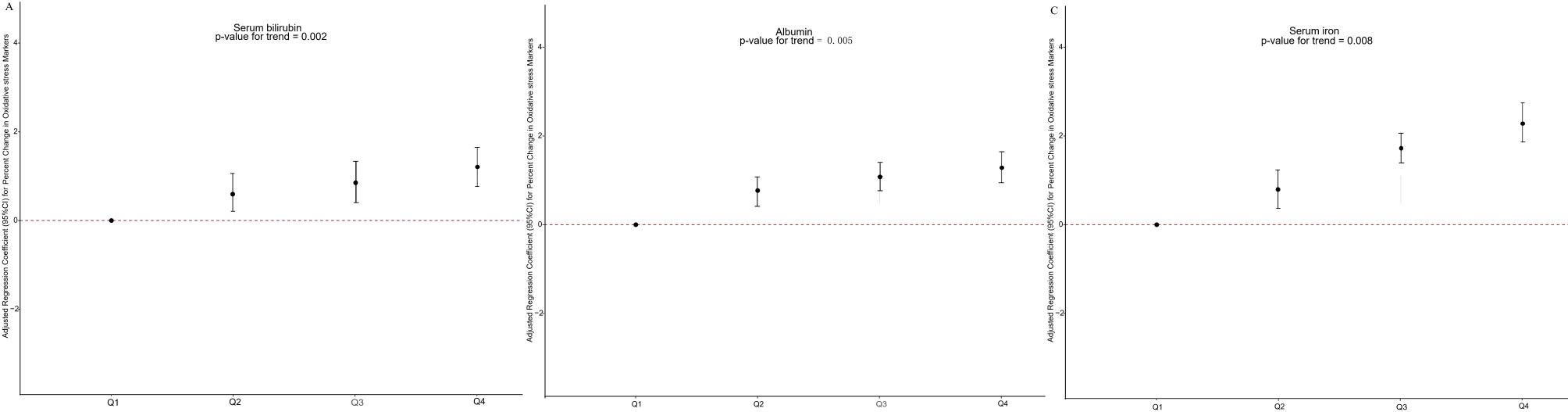


**Figure S8** Percent Change in Oxidative Stress Markers Associated with Increasing C-DII among Study Participants in model 2 for (a) serum bilirubin, (b) albumin, (c) serum iron. Model 2 adjusted for age, sex, race/ethnicity, smoking status, BMI, energy intake and poverty-income ratio.


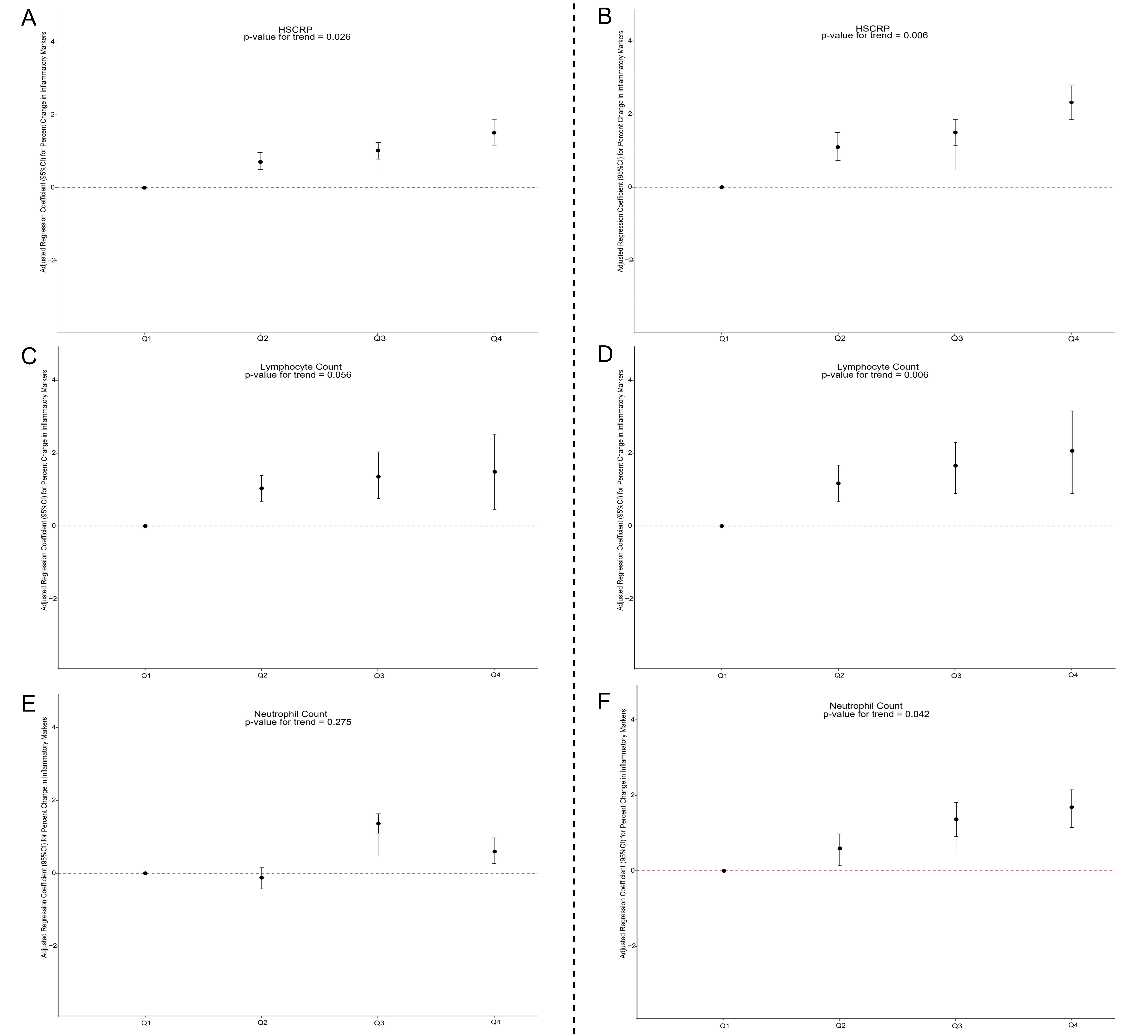


**Figure S9** Percent Change in Inflammation Markers Associated with Increasing C-DII among Study Participants in model 3 in different subgroups: (A、C、E) normal weight subgroup; (B、D、F) Overweight/obesity subgroup. Model 3 adjusted for age, sex, race/ethnicity, smoking status, BMI, energy intake, poverty-income ratio, body fat percentage and energy intake.


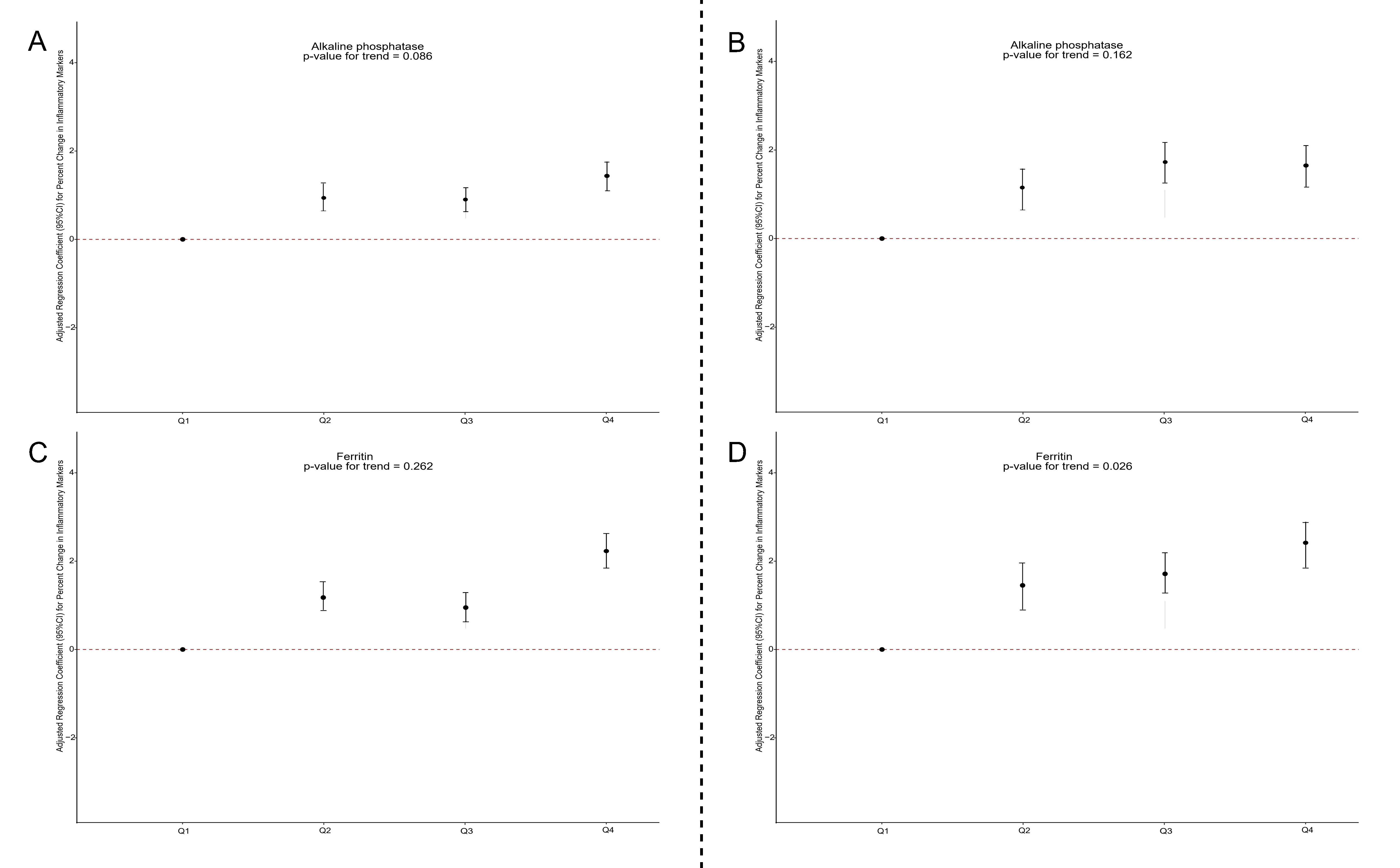


**Figure S10** Percent Change in Inflammation Markers Associated with Increasing C-DII among Study Participants in model 3 in different subgroups: (A、C) normal weight subgroup; (B、D) Overweight/obesity subgroup. Model 3 adjusted for age, sex, race/ethnicity, smoking status, BMI, energy intake, poverty-income ratio, body fat percentage and energy intake.


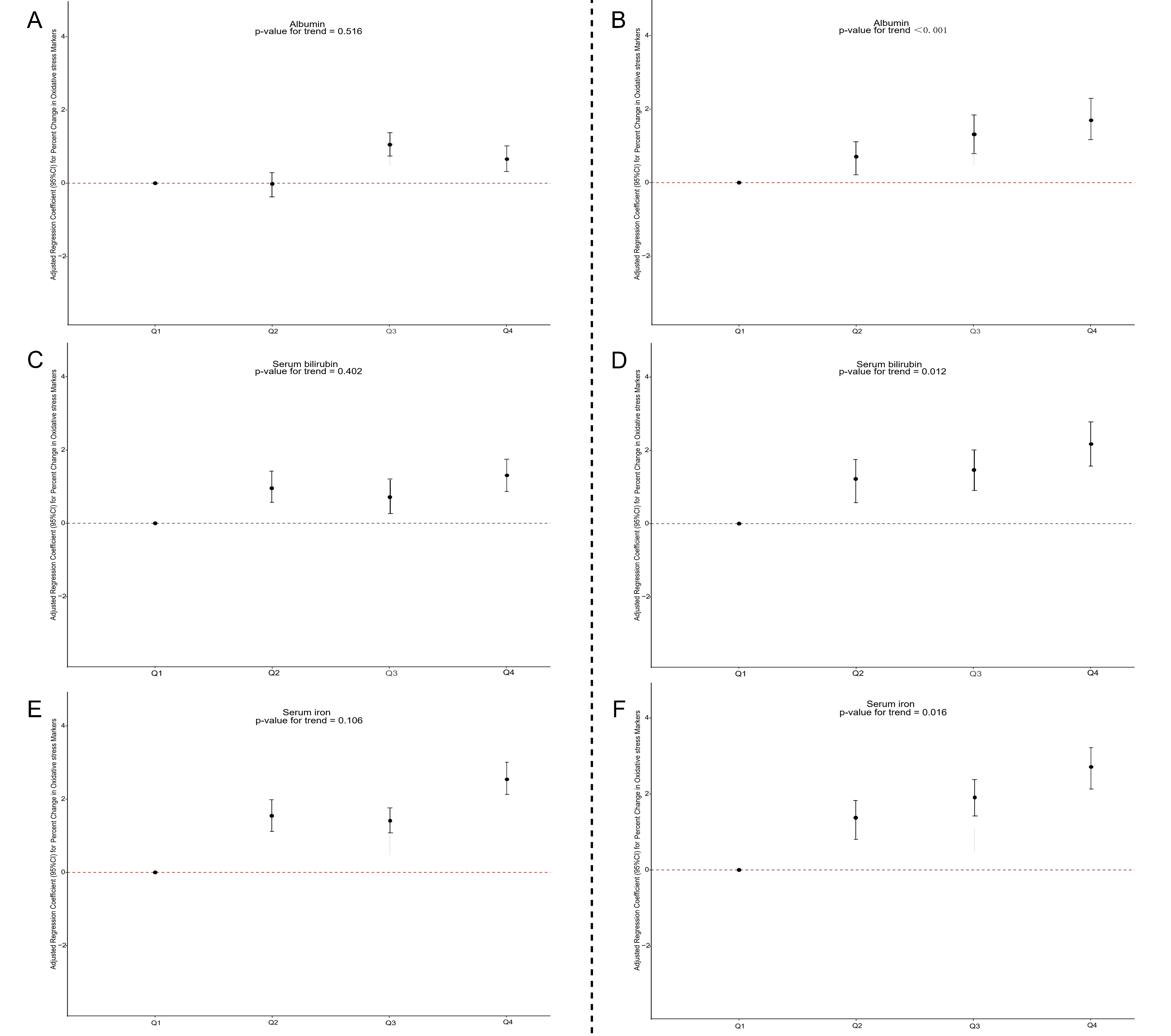


**Figure S11** Percent Change in Oxidative Stress Markers Associated with Increasing C-DII among Study Participants in model 3 in different subgroups: (A、C、E) normal weight subgroup; (B、D、F) Overweight/obesity subgroup. Model 3 adjusted for age, sex, race/ethnicity, smoking status, BMI, energy intake, poverty-income ratio, body fat percentage and energy intake.


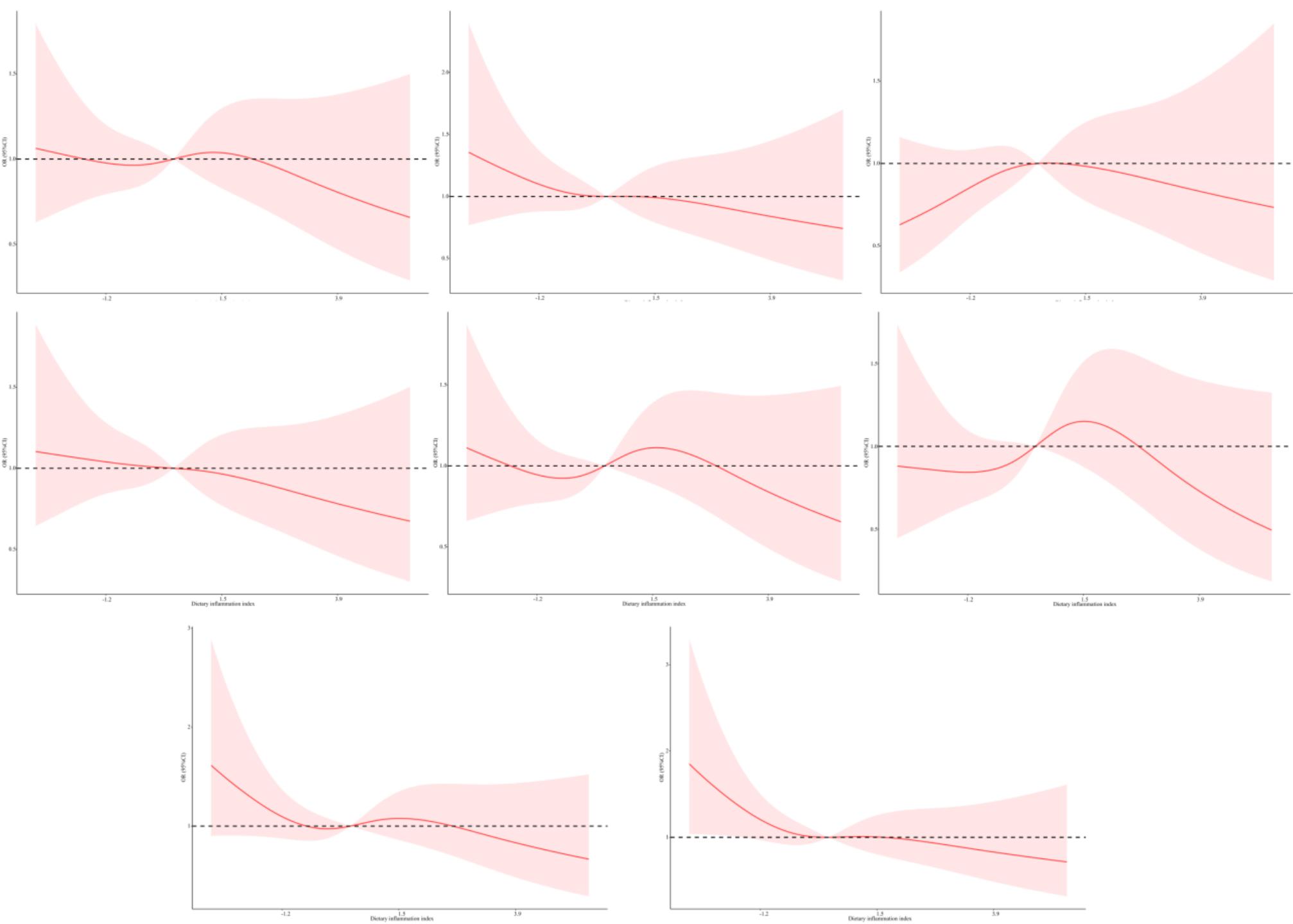


**Figure S12** Restricted cubic spline regression in unweighted data for (a) lymphocyte count, (b) neutrophil count, (c) ferritin (d) CRP, (e) alkaline phosphatase, (f) serum bilirubin, (g) albumin, (h) serum iron. The red line and area represent the estimated OR values and their corresponding 95% CI. Model adjusted for age, sex, race/ethnicity, smoking status, BMI, energy intake, poverty-income ratio, body fat percentage and energy intake.
